# Retraction Note: Injuries and outcomes resulting due to falls in elderly patients presenting to the emergency department of a tertiary care hospital – a cohort study

**DOI:** 10.1186/s12873-023-00867-x

**Published:** 2023-08-10

**Authors:** Salman Muhammad Soomar, Zeyanna Dhalla

**Affiliations:** 1 Department of Emergency Medicine, Aga Khan University, Karachi, Pakistan; 2 University of Michigan, Ann Arbor, MI USA

**Retraction Note: BMC Emergency Medicine (2023) 23:14**

10.1186/s12873-023-00784-z

The Editor has retracted this article at the corresponding author’s request. After the publication of this article, Aga Khan University undertook an inquiry, which revealed that the data presented in this article does not belong to the corresponding author, namely Salman Muhammad Soomar and the study dates mentioned in the article are incorrect. Additionally, the corresponding author has failed to provide a valid ethical approval document. Zeyanna Dhalla stated that she was unaware of the data ownership of this article. All authors agree to this retraction.

